# Self-perception of the Immediate Impact on the Voice of Gospel Singers after a one-hour Presentation

**DOI:** 10.1590/2317-1782/20232023002en

**Published:** 2023-11-20

**Authors:** Marluce dos Santos Maia Rodrigues, Fabiana Copelli Zambon, Claudia de Oliveira Lima Camargo Pacheco, Mara Suzana Behlau

**Affiliations:** 1 Centro de Estudos da Voz – CEV, São Paulo (SP), Brasil.

**Keywords:** Voice, Clinical Protocols, Speech Language and Hearing Sciences, Singing, Voice Disorders

## Abstract

**Purpose:**

To investigate the immediate impact on the voice of gospel singers with and without vocal complaints after a one-hour individual presentation.

**Methods:**

Application of an online questionnaire that addressed the following aspects: 1 - Sociodemographic data; 2 – Self-assessment of the ability to sing using the Evaluation of the Ability to Sing Easily (EASE-BR) protocol; 3 - Self-assessment of vocal fatigue symptoms using the Vocal Fatigue Index (VFI) protocol; and 4 - Self-assessment of voice handicap using the Voice Handicap Index 10 (VHI-10) protocol. Participants were divided into two groups: Group with Vocal Complaint (WVC) and Group with no Vocal Complaint (WnVC) based on the total score of the IDV-10. Data underwent descriptive and inferential statistical analysis with a significance level of 5%.

**Results:**

The study included 43 gospel singers with a median age of 34 years: 32 were in the WnVC group and 11 were in the WVC group. The WVC group reported hoarseness and experienced more difficulty while singing in the EASE, resulting in higher scores in both the VHI-10 and VFI protocols. A positive correlation was observed between singing difficulty and vocal handicap due to fatigue in amateur gospel singers, with this correlation being stronger within the WnVC group.

**Conclusion:**

After one hour of performance, singers with vocal complaints exhibited higher rates of vocal fatigue, vocal disadvantage, and greater difficulty in singing. Singers without complaints may have their ability to sing impaired by vocal fatigue. Variations in singing ability and vocal handicaps in amateur gospel singers may be directly related to vocal fatigue.

## INTRODUCTION

Gospel music is considered a form of worship to God by Christians. It emerged in the early spiritual movements of the 1850s, evolving into the popular style we know today. It can be performed both individually and in groups^(1)^. Because it is a devotional form of singing, this style is practiced with great intensity, with the need for a strong vocal technique to minimize vocal risks and ensure voice preservation^(1,2)^.

Contemporary Christian singers and worship leaders are considered a subset of contemporary commercial music and face vocal demands and vocal risks inherent to this style and musical genre^(2)^. Singers can be either amateurs or professionals, the main difference between amateur or volunteer singers is the pleasure and love of singing, not relying on it for their source of income. While amateur singers heavily rely on their voices, they often lack awareness of vocal health and hygiene, performing without the necessary guidance or resources to prevent issues or to access appropriate professional assistance^(2,3)^. Thus, speech-language pathologists (SLP) can assist this population in vocal training and rehabilitation, as well as in promoting vocal health and preventing voice problems^(4)^.

Since they generally lack proper guidance and support, these professionals may exhibit signs of fatigue, and subsequent vocal disadvantage^(3,5,6)^, which are common symptoms among amateur singers and may be related to high vocal demand^(5,7)^. Specifically, vocal fatigue is a kinesthetic symptom and it is self-reported by the individual^(7)^.

Vocal assessment is multi-dimensional; in addition to perceptual-auditory judgment and acoustic analysis, the person’s self-assessment is an easy evaluation that provides valuable information about a vocal problem and its impact on daily life^(8)^. Therefore, vocal self-assessment protocols play a significant role in the clinic, helping the patient in his self-perception of the dysphonia impact on their life, both socially and professionally. Moreover, they contribute to treatment adherence^(9)^.

According to the patient's vocal complaint, symptoms, and professional activity, the SLP may use different self-assessment tools to complement the voice assessment. Some examples of these self-assessment tools are the Evaluation of the Ability to Sing Easily (EASE)^(10)^, which evaluates singing ability after a vocal performance.; the Vocal Fatigue Index (VFI)^(5)^, which identifies vocal fatigue symptoms; and, the Voice Handicap Index with 10 items (VHI-10)^(11)^, a sensitive protocol for screening the presence or absence of dysphonia.

A previous investigation of vocal fatigue among professional opera singers identified an increased difficulty in singing middle and low notes after an extended period of singing^(12)^. A comparison of self-perceived vocal fatigue and the use of singing voice during the COVID-19 pandemic among professional and amateur singers indicated that professional singers had higher vocal fatigue scores, greater vocal demand, and a higher frequency of vocal training. Singers with vocal complaints had higher vocal fatigue scores, regardless of whether they were amateurs or professionals^(13)^.

Amateur gospel singers are often self-taught, and this population's vocal demand, without proper vocal guidance and preparation, can lead to immediate and long-term negative vocal impacts^(3,5,6)^. This population has specific vocal characteristics and demands. Therefore, the importance of researching their vocal practices and their immediate impact on performance becomes evident, as vocal disadvantage and fatigue can potentially have a negative effect on these singers' performances. Hence, this research aimed to examine the immediate impact on the voices of gospel singers, both those with and without vocal complaints, following a one-hour solo performance.

## METHOD

This is a quantitative cross-sectional field study, submitted to and approved by the Research Ethics Committee, under CAAE number 61330322.8.0000.8118 and protocol number 5.625.059. A questionnaire was completed online by amateur gospel singers immediately after a one-hour performance. Data collection took place in September and October 2022.

Participants were invited through social media announcements and by email sent to religious leaders and/or institutional emails of evangelical churches. Additionally, snowball sampling was employed; this is a non-probabilistic sampling form that considers a network of references and referrals. The inclusion criteria were as follows: being a solo gospel singer, between 18 and 55 years old, and performing in evangelical churches in Brasília, Brazil. All participants agreed to participate in the study and signed the Informed Consent Form. Exclusion criteria were as follows: having received voice therapy in the past, having a cold or upper respiratory tract infection on the day of data collection, or not completing the questionnaire.

Participants were divided into two groups: With Vocal Complaint Group (WVC) and With no Vocal Complaint Group (WnVC). Group assignment was based on the VHI-10 score. The VHI-10 is a sensitive instrument for identifying potential vocal handicaps; thus, participants who scored above the protocol's cutoff score (7.5) were placed in the WVC group.

The questionnaire, developed and distributed to participants through a free and online virtual platform, was self-administered, and researchers were not present during its completion. Participants received written instructions to complete the questionnaire immediately after a one-hour solo performance. The singers' performances happened on different days and locations during the data collection period; however, all performances were sixty minutes long.

All questions were presented in a single document divided into four parts: 1 - Sociodemographic Data; 2 - Self-assessment of singing using the Evaluation of the Ability to Sing Easily, Brazilian Portuguese version (EASE-BR); 3 - Self-assessment of vocal fatigue using the Vocal Fatigue Index in the Brazilian Portuguese version (VFI); and 4 - Self-assessment of vocal handicap and vocal complaints using the Voice Handicap Index with 10 items in the Brazilian Portuguese version (VHI-10).

The EASE scale was specifically developed for singers, and it is particularly attuned to the unique characteristics of this population's voice^(10)^. This scale was translated and culturally adapted for Brazilian Portuguese as EASE-BR^(14)^. The scale has 22 questions with four response options on a Likert scale based on the frequency of the described situation. The score is the sum of the 19 negative items, where 0 = no, 1 = mildly, 2 = moderately, and 3 = extremely, along with the three positive items (questions 6, 12, and 21) which are reverse scored.

The Vocal Fatigue Index (VFI)^(15)^ was translated^(16)^ and validated^(5)^ for the Brazilian Portuguese; it is sensitive in identifying vocal fatigue signs. The VFI consists of 17 questions that are divided into four factors: factors: 1 - tiredness and voice impairment (questions 1 to 7); 2 – voice avoidance (questions 8 to 10); 3 - physical discomfort associated with voicing (questions 11 to 14); 4 - improvement of voice symptoms with rest (questions 15 to 17). Each question is rated on a Likert scale from 0 to 4, 0 = never, 1 = almost never, 2 = sometimes, 3 = almost always, 4 = always. The protocol score is the total sum of factors 1, 2, and 3, and the inverse value of factor 4. Higher scores in factors 1, 2, and 3, indicate more severe vocal fatigue symptoms, while in factor 4, a higher score indicates better vocal recovery. The total factor is calculated using the following formula: Total Factor = Factor 1 + Factor 2 + Factor 3 + (12 - Factor 4); the protocol cutoff score is 11.5 points. The VFI was chosen for this study as it is an effective tool for assessing vocal fatigue in various populations, including singers^(13)^, and this symptom can affect both professionals and amateurs. However, the VFI is not specific to singers, thus the EASE-BR was also used.

The VHI-10 is a reduced version of a protocol originally with 30 questions^(17)^. The VHI-10 has been validated in Brazilian Portuguese^(11)^, and it is sensitive to distinguish dysphonic from non-dysphonic individuals. Therefore, it was chosen as the instrument to categorize singers into groups with and without vocal complaints. The VHI-10 has 10 questions that should be answered on a 5-point Likert scale, where 0 = never and 4 = always. The total score is calculated by the simple sum of the responses, where 0 indicates no vocal handicap and 40 indicates the maximum handicap; the cutoff score is 7.5 points.

There were 43 gospel singers, with a median age of 34 years old; 21 (48.84%) were female, and 22 (51.16%) were male. They were divided into two groups: WnVC – 32 (74.42%) gospel singers, 18 men and 15 women; WVQ – 11 (25.58%) gospel singers, five men and six women.

Most participants (N=29; 67.44%) completed higher education, while 7 (16.28%) were currently working on their bachelor's, and 5 (11.63%) were in high school. A total of 31 participants (72.09%) considered themselves voice professionals; however, only 18 (41.86%) performed vocal warm-up and/or cool-down. Only 8 participants (16.60%) took singing lessons, with a median of one-hour lessons per week. The median singing experience was 20 years. None of the participants reported seeking vocal healthcare.

The method used to categorize the groups with and without vocal complaints was the VHI-10, a validated instrument that utilizes established cutoff point standards for this purpose. Most participants (N=32) did not score above the VHI-10 cutoff point, and 11 were classified as having vocal handicaps, reporting various vocal complaints.

Data were analyzed descriptively and inferentially using SPSS 25.0 software. The significance level was set at 5% for inferential analyses.

In the descriptive analysis of quantitative variables, measures of central tendency (mean and median), variability (standard deviation), and position (minimum, maximum, first quartile, and third quartile) were calculated, along with absolute frequency and relative percentage frequency.

Inferential analysis of the association between nominal qualitative variables was performed using the Chi-Square test. A comparison of non-normally distributed quantitative variables and ordinal qualitative variables between two independent groups was performed using the Mann-Whitney test. The correlation between non-normally distributed quantitative variables and ordinal qualitative variables was assessed using the Spearman Correlation Test, with values ranging from r=0.10 to 0.30 considered weak correlation, r=0.40 to 0.6 considered moderate, and r=0.70 to 1 indicative of a strong correlation^(18)^.

## RESULTS

Pearson's Chi-Square test revealed an association between the WnVC group and the absence of hoarseness complaints, and the WVC group and the presence of hoarseness complaints (p=0.043). The Mann-Whitney test revealed that the group with vocal complaints had higher mean scores in all protocols. See Table 1.

**Table 1:** Inferential analysis comparing the variables EASE-BR, VFI, and VHI-10 considering the group of gospel singers

Variable	GROUP	Mean	SD	Median	U	p-value
VHI-10	SQ	1.88	1.83	1.50	0.000	<0.001*
	CQ	10.36	2.58	10.00		
EASE-Br	SQ	17.53	9.27	16.50	61.000	0.001*
	CQ	27.82	8.83	25.00		
VFI						
Tiredness and voice impairment	SQ	22.31	9.84	21.50	36.000	<0.001*
	CQ	38.91	9.44	37.00		
VFI						
Voice avoidance	SQ	1.78	1.58	1.50	44.500	<0.001*
	CQ	4.55	1.92	4.00		
VFI						
Physical discomfort associated with voicing	SQ	1.41	2.26	0.00	122.500	0.105
	CQ	2.91	2.98	2.00		
VFI						
Improvement of voice symptoms with rest	SQ	8.28	4.12	10.00	167.000	0.798
	CQ	8.55	2.98	9.00		
VFI						
Total	SQ	29.22	12.56	28.00	36.000	<0.001*
	CQ	49.82	11.03	50.00		

Mann-Whitney Test

* statistically significant variables

**Caption:** SD=standard deviation; U=Mann-Whitney U value

For all gospel singers, there was a strong positive correlation between EASE-BR and VFI in the tiredness and voice impairment factor (p<0.001), a strong correlation between EASE-BR and VFI total score (p<0.001), a moderate correlation between EASE-BR and VFI in the voice avoidance factor (p<0.001), a moderate correlation between VHI-10 and VFI in the tiredness and voice impairment factor (p<0.001), a moderate correlation between VHI-10 and VFI in the vocal avoidance factor (p<0.001), a weak correlation between VHI-10 and VFI in the physical discomfort associated with voicing factor (p=0.012), and a moderate correlation between VHI-10 and VFI total score in gospel singers (p<0.001).

For the WVC group, there was a strong positive correlation between EASE-BR and VFI in the tiredness and voice impairment factor (p<0.001) and a strong correlation between EASE-BR and VFI total score (p=0.002). On the other hand, the WnVC group presented a strong positive correlation between EASE-BR and VFI in the tiredness and voice impairment factor (p<0.001), a strong correlation between EASE-BR and VFI total score (p<0.001), a moderate correlation between EASE-BR and VFI in the voice avoidance factor (p=0.017), and a moderate correlation between VHI-10 and VFI voice avoidance factor (p=0.017). Table 2 summarizes the correlation between the VFI protocol and EASE-BR and VHI-10.

**Table 2:** EASE-BR and VHI-10 correlations with the VFI in gospel singers, gospel singers with vocal complaints, and gospel singers without vocal complaints

		Total		WnVC		WVC	
		EASE-Br	VHI-10	EASE-Br	VHI-10	EASE-Br	VHI-10
VFI Tiredness and voice impairment	r	0.948	0.545	0.931	0.154	0.888	0.379
	p-value	<0.001*	<0.001*	<0.001*	0.399	<0.001*	0.250
VFI Voice avoidance	r	0.576	0.645	0.420	0.418	0.589	0.238
	p-value	<0.001*	<0.001*	0.017*	0.017*	0.057	0.482
VFI Physical discomfort associated with voicing	r	0.168	0.379	0.068	0.306	-0.166	0.555
	p-value	0.283	0.012*	0.713	0.088	0.626	0.077
VFI Improvement of voice symptoms with rest	r	-0.081	0.085	-0.100	0.141	0.083	0.473
	p-value	0.605	0.586	0.585	0.442	0.808	0.142
VFI total	r	0.887	0.537	0.840	0.134	0.817	0.414
	p-value	<0.001*	<0.001*	<0.001*	0.466	0.002*	0.206

Spearman Correlation Test

* statistically significant variables

**Caption:** r=correlation coefficient

There was a difference in the VHI-10 scores with tiredness and voice impairment factor (U=148.5; p=0.045), EASE-BR (U=138; p=0.024), and VFI total score (U=121.5; p=0.008) and women presented higher values than men. However, for the VFI improvement of voice symptoms with rest factor, men had higher values than women (U=143; p=0.029), as shown in Table 3.

**Table 3:** Inferential analysis comparing the EASE-BR, VFI, and VHI-10 by gender variable in gospel singers

Variable	Gender	Mean	SD	Median	U	p-value
VHI-10	Male	3.55	3.84	2.50	202.000	0.477
	Female	4.57	4.69	3.00		
EASE-Br	Male	16.77	9.31	17.00	138.000	0.024*
	Female	23.71	9.92	21.00		
VFI						
Tiredness and voice impairment	Male	22.82	10.94	23.00	148.500	0.045*
	Female	30.48	12.25	31.00		
VFI						
Voice avoidance	Male	2.09	1.77	2.00	183.000	0.234
	Female	2.90	2.28	2.00		
VFI						
Physical discomfort associated with voicing	Male	1.27	1.83	0.00	200.500	0.420
	Female	2.33	3.02	0.00		
VFI						
Improvement of voice symptoms with rest	Male	9.59	3.14	10.00	143.000	0.029*
	Female	7.05	4.12	8.00		
VFI						
Total	Male	28.59	12.38	26.50	121.500	0.008*
	Female	40.67	15.48	39.00		

Mann-Whitney Test

* statistically significant variables

**Caption:** SD=standard deviation; U=Mann-Whitney U value

The females from the WnVC group presented a higher value in the EASE-BR (U=73; p=0.044) and the VFI total score (U=71; p=0.036). However, males presented higher values of the VFI improvement of voice symptoms with rest factor (U=75.5; p=0.049), as shown in Table 4. No difference was observed between the protocol scores and the gender of individuals in the WVC group.

**Table 4:** Inferential analysis comparing the EASE-BR, VFI, and VHI-10 by gender in gospel singers without vocal complaints

Variable	Gender	Mean	SD	Median	U	p-value
VHI-10	Male	2.06	2.18	1.50	122.500	0.892
	Female	1.64	1.28	1.50		
EASE-Br	Male	14.67	8.08	12.50	73.000	0.044*
	Female	21.21	9.66	17.50		
VFI						
Tiredness and voice impairment	Male	19.94	9.28	19.50	87.000	0.138
	Female	25.36	10.02	22.00		
VFI						
Voice avoidance	Male	1.83	1.82	1.00	117.500	0.738
	Female	1.71	1.27	2.00		
VFI						
Physical discomfort associated with voicing	Male	1.06	1.83	0.00	112.000	0.549
	Female	1.86	2.71	0.00		
VFI						
Improvement of voice symptoms with rest	Male	9.56	3.42	10.00	75.500	0.049*
	Female	6.64	4.48	7.00		
VFI						
Total	Male	25.28	10.18	25.00	71.000	0.036*
	Female	34.29	13.84	34.50		

Mann-Whitney Test

* statistically significant variables

**Caption:** SD=standard deviation; U=Mann-Whitney U value

## DISCUSSION

Amateur gospel singers often perform with high vocal demand and without adequate vocal guidance and preparation, which can lead to vocal complaints, vocal fatigue, and vocal handicap^(3,5,6)^. Therefore, this study aimed to examine the immediate impact on the voices of gospel singers, both those with and without vocal complaints, following a one-hour solo performance.

The age range and years of experience indicate that this population typically begins their singing activities at a young age and often as self-taught individuals. Evangelical singers have been singing in their church for many years, and many begin their vocal activities in children's choirs with no formal singing lessons^(19)^.

Most participants reported being vocal professionals, such as teachers, journalists, musicians, religious leaders, etc. However, they did not report engaging in vocal health practices, such as warm-up or cool-down exercises. In addition to the demanding vocal requirements of singing in church, these amateur singers may also have a high vocal demand at work and may not have a proper vocal care routine or frequent visits to ear, nose, and throat (ENT) doctors^(2,3,20)^. Furthermore, no participants sought voice-related care, which may indicate that this concern is being neglected. The underappreciation of voice problem symptoms is also common among other groups of voice professionals, such as teachers and teleoperators^(21–24)^.

Both groups had mean VFI scores above the cutoff point; however, the group with vocal complaints presented even higher scores, as well as greater vocal handicap and greater difficulty in singing easily (Table 1). Furthermore, the EASE-BR and the VHI-10 protocols indicated hoarseness as a prevalent symptom in this group that continues to sing despite their vocal complaints, which can worsen their laryngeal condition. A previous study that evaluated vocal disadvantage in 206 popular singers revealed that those with vocal complaints reported a lower self-perceived quality of life and more significant vocal issues^(25)^.

Especially for singers with no vocal complaints, difficulties related to singing ability were associated with restriction caused by vocal fatigue, which is probably a consequence of the high vocal demand for gospel singing (Table 2). A similar result was noted in a controlled study involving 30 adult choir singers. Even though these singers did not initially report any vocal complaints, as fatigue levels increased, so did the number of vocal complaints^(26)^. Although the participants with no vocal complaints scored below the VHI-10 cutoff point, the complaints highlighted by this protocol seemed to be related to the restriction caused by vocal fatigue symptoms.

For all groups, it was evident that vocal fatigue symptoms directly affect the ability to sing and can contribute to a greater perception of vocal handicap. "It is known that vocal fatigue can make singing difficult by altering the vocal range and its transition regions^(12)^. These data suggest that this population can benefit from vocal guidance emphasizing vocal warm-up and cool-down techniques. These practices help prepare the vocal musculature for the demands and promote a balanced vocal production^(27)^. Additionally, cool-down reduces vocal strain and helps remove lactic acid, preventing muscle pain and fatigue^(28)^.

The comparison between men and women, shown in Tables 3 and 4, shows that women have greater variability in singing ability as well as handicap, both of which are related to symptoms of vocal fatigue. On the other hand, men tend to have more complaints related to vocal fatigue recovery. This could be explained by women experiencing greater vocal disadvantage compared to men due to specific anatomical and physiological characteristics in females, such as the shape of the cricoid and thyroid cartilages, which often lead to a physiological gap^(29)^ in female larynges. Also, vocal fatigue can negatively impact the singing ability^(12,26)^.

One of the present study limitations is the fact that the protocols were applied only immediately after the performance, hence, it was not possible to assess any complaints and symptoms that may have been present before the performance. In addition, no questions regarding overall health and singing lessons were asked. Furthermore, sample size calculations were not performed; therefore, the inferences in this study are specific to the studied group. It is noteworthy that the Modern Singing Handicap Index (MSHI)^(30)^ could have been employed to identify specific complaints within this population and to compare them with the vocal handicap reported in other studies. However, it should be noted that this instrument does not yet have a defined cutoff score. Nevertheless, we were able to identify certain specific singing voice complaints using the EASE-BR protocol.

The present study outcomes have important implications for the SLP clinical practice with gospel singers. The results indicate that these singers, even with no vocal complaints, may present vocal fatigue and handicap after a performance. This underscores the significance of offering appropriate vocal guidance and vocal health prevention and promotion programs for this population. The use of vocal self-assessment protocols, such as EASE-BR, VHI-10, and VFI, can be valuable in identifying vocal fatigue and handicaps to inform SLP interventions.

## CONCLUSION

Singers with vocal complaints have higher vocal fatigue levels, vocal disadvantage, and greater difficulty in singing immediately after a one-hour performance. Singers without vocal complaints may find their singing ability affected by symptoms of vocal fatigue. Variations in the singing ability and vocal disadvantages among amateur gospel singers may be directly related to the vocal fatigue perception.

Further studies are required to explore the unique requirements of this population and to suggest vocal preparation strategies for these individuals. In addition, these singers would benefit from vocal health promotion and prevention measures, as they are self-taught and face high vocal demands in their church settings.
