# Supplementary material for: Self-perception of the Immediate Impact on the Voice of Gospel Singers after a one-hour Presentation
Source: Codas. 2023 Nov 20;36(2):e20230002. doi: 10.1590/2317-1782/20232023002en (PMC10715635; doi:10.1590/2317-1782/20232023002en)
Supplement: Translation

# Translation: Autopercepção do Impacto Imediato na Voz de Cantores Gospel após uma Apresentação de uma hora

**DOI:** 10.1590/2317-1782/20232023002pt

**Authors:** Marluce dos Santos Maia Rodrigues, Fabiana Copelli Zambon, Claudia de Oliveira Lima Camargo Pacheco, Mara Suzana Behlau

**Affiliations:** 1 Centro de Estudos da Voz – CEV, São Paulo (SP), Brasil.

**Keywords:** Voz, Protocolos, Fonoaudiologia, Canto, Distúrbios da Voz

## Abstract

**Objetivo:**

Investigar o impacto imediato na voz de cantores gospel com e sem queixa vocal após uma apresentação individual de uma hora.

**Método:**

Aplicação de um questionário online que abordou os seguintes aspectos: 1 - Dados sociodemográficos; 2 – Autoavaliação da habilidade de cantar pelo protocolo Evaluation of the Ability to Sing Easily (EASE-BR); 3 - Autoavaliação de sintomas de fadiga vocal pelo Índice de Fadiga Vocal (IFV); e 4 - Autoavaliação da desvantagem vocal pelo protocolo Índice de Desvantagem Vocal 10 (IDV-10). Os participantes foram divididos em dois grupos: Grupo Com Queixa (CQ) e Grupo Sem Queixa (SQ) com base no escore total do IDV-10. Os dados passaram por análise estatística descritiva e inferencial considerando o nível de significância de 5%.

**Resultados:**

Participaram 43 cantores gospel com idade mediana de 34 anos, divididos entre 32 do grupo SQ e 11 do grupo CQ. O grupo CQ autorrelatou rouquidão e maior dificuldade ao cantar no EASE e maiores escores nos protocolos IDV-10 e IFV. Foi evidenciada correlação positiva entre a dificuldade ao cantar e a desvantagem vocal com a fadiga de cantores amadores gospel, sendo que essa correlação foi maior para o grupo SQ.

**Conclusão:**

Cantores com queixa vocal apresentaram maiores índices de fadiga vocal, desvantagem vocal e maior dificuldade para cantar após uma hora de apresentação. Cantores sem queixa podem ter a habilidade de cantar prejudicada pela fadiga vocal. Variações na habilidade ao cantar e desvantagens vocais de cantores amadores gospel podem ter relação direta com a fadiga vocal.

## INTRODUÇÃO

A música gospel é considerada uma música de adoração a Deus pelos cristãos, surgiu nos primeiros movimentos espirituais da década de 1850, evoluindo para o estilo popular dos dias de hoje e pode ser cantada individualmente ou em grupo^(1)^. Por ser um canto devocional, este estilo é praticado com muito vigor, sendo necessária uma técnica vocal sólida, a fim de se diminuir riscos e prevenir alterações vocais^(1,2)^.

Cantores cristãos contemporâneos e líderes de louvor são considerados um subconjunto da música comercial contemporânea e enfrentam demandas e riscos vocais inerentes a este estilo e gênero musical^(2)^. Cantores podem ser amadores ou profissionais, sendo que o que diferencia esses dois grupos é que o amador ou voluntário canta somente pelo prazer e amor ao canto, não fazendo desta prática o seu sustento.

Cantores amadores, apesar de dependerem muito de sua voz, podem não ter a consciência sobre saúde e higiene vocal e geralmente atuam sem um direcionamento ou orientação vocal adequada para evitar problemas ou buscar os meios e profissionais adequados para solucioná-los^(2,3)^. Deste modo, o fonoaudiólogo pode auxiliar essa população na habilitação e reabilitação vocal, bem como na promoção de saúde e prevenção de problemas de voz^(4)^.

Por geralmente não terem um acompanhamento e orientações adequadas, esses profissionais podem apresentar sinais de fadiga e consequente desvantagem vocal^(3,5,6)^, sintomas comuns em cantores amadores, podendo estar relacionados com a alta demanda vocal^(5,7)^. A fadiga vocal, especificamente, é um sintoma de caráter cinestésico e autorrelatado pelo indivíduo^(7)^.

A avaliação vocal é multidimensional e além da avaliação perceptivoauditiva e acústica, a autoavaliação do sujeito é de fácil aplicação e pode fornecer informações importantes sobre um problema vocal e o seu impacto no cotidiano^(8)^. Deste modo, os protocolos de autoavaliação vocal têm uma grande importância na área clínica, ajudando o paciente na autopercepção do impacto da disfonia em sua vida, seja na área social ou profissional, mas sobretudo contribuindo também para a adesão ao tratamento^(9)^.

A depender do tipo de queixa, sintomas apresentados e profissão do cliente, o fonoaudiólogo pode utilizar estes instrumentos para complementar a avaliação vocal. Dentre os protocolos, destacam-se a escala *Evaluation of the Ability to Sing Easily* (EASE)^(10)^, para autoavaliação da habilidade de cantar, usada após uma apresentação; o Índice de Fadiga Vocal (IFV)^(5)^, para identificar sintomas de fadiga vocal e o Índice de Desvantagem Vocal 10 (IDV-10)^(11)^, protocolo que é sensível para rastrear presença ou ausência de disfonia.

Uma investigação de fadiga vocal em cantoras de ópera profissionais identificou aumento da dificuldade ao cantar notas médias e graves após um período prolongado de canto^(12)^. A comparação da autopercepção da fadiga vocal e o uso da voz cantada durante a pandemia de COVID-19 entre cantores profissionais e amadores, indicou que cantores profissionais tinham escores mais altos de fadiga e apresentavam maior demanda vocal e maior frequência de treinamento vocal. Cantores com queixas vocais tiveram escores mais altos de fadiga vocal, independente se amadores ou profissionais^(13)^.

Cantores amadores gospel muitas vezes são autodidatas e a demanda vocal dessa população, sem orientação e preparação vocais adequadas, pode causar impactos vocais negativos imediatamente após a performance e também a longo prazo^(3,5,6)^. Essa população possui características vocais e demandas distintas e, nessa perspectiva, fica evidente a necessidade de investigar suas práticas vocais bem como o impacto imediato da performance, pois acredita-se que a desvantagem e a fadiga vocal tenham um impacto negativo no rendimento desses cantores. Deste modo, o objetivo desta pesquisa foi investigar o impacto imediato na voz de cantores gospel com e sem queixa vocal após uma apresentação individual de uma hora.

## MÉTODO

Trata-se de estudo quantitativo transversal, de campo, submetido e aprovado pelo Comitê de Ética em Pesquisa, sob CAAE nº 61330322.8.0000.8118 e parecer nº 5.625.059. Um questionário foi respondido de forma *online* por cantores amadores gospel imediatamente após uma apresentação de uma hora. A coleta de dados foi realizada nos meses de setembro e outubro de 2022.

Os participantes foram recrutados via divulgação em redes sociais e por e-mail enviados para líderes religiosos e/ou e-mails institucionais de igrejas evangélicas. Além disso, foi utilizada a amostragem em *snow-ball*, uma forma de amostra não probabilística que usa rede de referências e indicações. Os critérios de inclusão foram: ser cantor solo de música gospel, ter entre 18 e 55 anos e atuar em igrejas evangélicas de Brasília/DF. Todos os participantes concordaram em participar do estudo e assinaram o termo de consentimento livre e esclarecido. Os critérios de exclusão foram: já ter realizado fonoterapia na área da voz, estar gripado ou com infecções de vias aéreas superiores no dia da coleta ou não responder o questionário na íntegra.

Os participantes foram divididos em dois grupos: Grupo Com Queixa (CQ) e Grupo Sem Queixa (SQ). A divisão dos grupos foi realizada com base no escore total do protocolo de autoavaliação IDV-10 respondido pelos cantores. O IDV-10 é um instrumento sensível para evidenciar possíveis queixas vocais e, assim, os cantores que obtiveram escore superior à nota de corte do protocolo (7,5) foram alocados no grupo CQ.

O questionário, elaborado e enviado aos participantes por meio de uma plataforma virtual gratuita e *online*, era autoaplicável e os pesquisadores não estavam presentes para aplicação, os participantes receberam orientações por escrito para responderem o questionário imediatamente após uma apresentação individual de uma hora. As apresentações dos cantores ocorreram em dias e locais diferentes durante o período da coleta, mas todas tiveram a mesma duração, de sessenta minutos.

Todas as questões foram apresentadas em um único documento que foi dividido em quatro partes: 1 - Dados sociodemográficos; 2 - Autoavaliação do canto pela escala *Evaluation of the Ability to Sing Easily* (EASE-BR); 3 - Autoavaliação da fadiga vocal pelo Índice de Fadiga Vocal (IFV) e; 4 - Autoavaliação da desvantagem e queixas vocais pelo Índice de Desvantagem Vocal 10 (IDV-10).

A escala EASE foi desenvolvida especificamente para cantores, sendo particularmente sensível à essência da voz dessa população^(10)^. Essa escala foi traduzida e culturalmente adaptada para o português brasileiro como EASE-BR^(14)^. Essa escala contém 22 questões e quatro alternativas em uma escala *likert* de acordo com frequência de ocorrência da situação descrita e o escore é obtido por meio do somatório dos 19 itens negativos sendo 0 = não, 1 = um pouco, 2 = moderadamente e 3 = muito; com os três itens positivos (questões 6, 12 e 21) somados de forma reversa.

O IFV^(15)^, foi traduzido^(16)^ e validado^(5)^ para o português brasileiro e é sensível para identificar sinais de cansaço vocal. O IFV é composto de 17 questões que se dividem em: 1 - fadiga e limitação vocal (questões 1 a 7); 2 - restrição vocal (questões 8 a 10); 3 - desconforto físico associado à voz (questões 11 a 14); 4 - recuperação com o repouso vocal (questões 15 a 17). Cada questão varia de uma escala *likert* de 0 a 4, sendo 0 = nunca, 1 = quase nunca, 2 = às vezes, 3 = quase sempre, 4 = sempre. O protocolo é calculado por meio de uma pontuação total, definido como a soma dos fatores 1, 2 e 3 e o valor invertido do Fator 4, visto que, nos fatores 1, 2 e 3, quanto maior o escore, maiores são os sintomas de fadiga e no 4, quanto maior o escore, melhor é a recuperação vocal. O fator total foi calculado usando a seguinte fórmula: Fator Total = Fator 1 + Fator 2 + Fator 3 + (12-Fator 4), a nota de corte do protocolo é de 11,5. O IFV foi escolhido para este estudo, pois demonstrou ser uma ferramenta eficaz para avaliar a fadiga vocal em diversas populações, incluindo cantores^(13)^, sendo este um sintoma que pode afetar tanto profissionais quanto amadores. Entretanto, sabe-se que o IFV não é específico para cantores, por isso optou-se por associá-lo a um protocolo específico (EASE-BR).

O IDV-10, é um protocolo que foi reduzido de uma versão mais extensa com 30 questões^(17)^. O IDV-10, validado no português brasileiro^(11)^, é sensível para rastrear sujeitos disfônicos e não-disfônicos e, por isso, este foi o instrumento escolhido para separar os cantores nos grupos com e sem queixas vocais. O IDV-10 possui 10 questões que devem ser respondidas numa escala *likert* de 5 pontos, sendo 0 nunca e 4 sempre, com cálculo do escore total através da somatória das respostas, sendo que 0 indica nenhuma desvantagem e 40, desvantagem máxima e nota de corte correspondente a 7,5 pontos.

Participaram do estudo 43 cantores gospel com mediana da idade de 34 anos, sendo 21 (48,84%) do sexo feminino e 22 (51,16%) do sexo masculino, divididos entre: SQ– 32 (74,42%) cantores gospel, sendo 18 homens e 15 mulheres; CQ – 11 (25,58%) cantores gospel, sendo cinco homens e seis mulheres.

Dos 43 cantores que participaram do estudo, a maioria (29 – 67,44%) tinha ensino superior completo, seguido de 7 (48,84%) que estavam cursando o ensino superior e 5 (11,63%) que cursavam o ensino médio. A maioria dos participantes (31 – 72,09%) se consideravam profissionais da voz, entretanto somente 18 (41,86%) realizavam algum procedimento de aquecimento e/ou desaquecimento vocal, e apenas 8 (16,60%) faziam aulas de canto, tendo mediana do tempo de aula de uma hora por semana. A mediana do tempo de canto foi de 20 anos. Nenhum dos participantes relatou ter buscado atendimento na área da Voz.

O método utilizado para divisão dos grupos com e sem queixa vocal foi um instrumento validado, o IDV-10, por ter um critério objetivo para essa categorização. A maioria dos participantes (N=32) não obteve pontuação acima da nota de corte no IDV-10 e 11 foram classificados como tendo desvantagem vocal, apresentando diversas queixas em relação a sua voz.

Os dados foram analisados de forma descritiva e inferencial utilizando-se o software SPSS 25.0. Foi considerado um nível de significância de 5% para as análises inferenciais.

Na análise descritiva das variáveis quantitativas foram calculadas as medidas de tendência central (média e mediana), variabilidade (desvio-padrão) e posição (mínimo, máximo, primeiro e terceiro quartis), com medidas de frequência absoluta e a frequência relativa percentual.

A análise inferencial de associação entre as variáveis qualitativas nominais foi realizada com o teste Qui-quadrado, a comparação das variáveis quantitativas não-normais e qualitativas ordinais entre dois grupos independentes foi realizada com o Teste de Mann-Whitney e a correlação entre as variáveis quantitativas não-normais e qualitativas ordinais foi realizada com o Teste de Correlação de Spearman, sendo considerados valores de r= 0,10 até 0,30 como correlação fraca; r = 0,40 até 0,6 como moderada e r = 0,70 até 1 como indicativo de forte correlação^(18)^.

## RESULTADOS

O teste Qui-Quadrado de Pearson evidenciou que houve associação entre o grupo SQ e a ausência de queixa de rouquidão, e o grupo CQ e a presença de queixa de rouquidão (p=0,043). O Teste de Mann-Whitney mostrou que o grupo com queixa vocal apresentou escores médios mais altos em todos os protocolos. Ver Tabela 1.

**Tabela 1:** Análise inferencial de comparação das variáveis EASE-Br, IFV e IDV-10 em função da variável grupo em cantores gospel

Variável	GRUPO	Média	DP	Mediana	U	p-valor
IDV-10	SQ	1,88	1,83	1,50	0,000	<0,001^*^
	CQ	10,36	2,58	10,00		
EASE-Br	SQ	17,53	9,27	16,50	61,000	0,001*
	CQ	27,82	8,83	25,00		
IFV						
Fadiga e limitação vocal	SQ	22,31	9,84	21,50	36,000	<0,001*
	CQ	38,91	9,44	37,00		
IFV						
Restrição vocal	SQ	1,78	1,58	1,50	44,500	<0,001*
	CQ	4,55	1,92	4,00		
IFV						
Desconforto físico associado à voz	SQ	1,41	2,26	0,00	122,500	0,105
	CQ	2,91	2,98	2,00		
IFV						
Recuperação com repouso vocal	SQ	8,28	4,12	10,00	167,000	0,798
	CQ	8,55	2,98	9,00		
IFV						
Total	SQ	29,22	12,56	28,00	36,000	<0,001*
	CQ	49,82	11,03	50,00		

Teste de Mann-Whitney

* variáveis estatisticamente significantes

**Legenda:** DP=desvio padrão; U= valor de U de Mann-Whitney

Para todos os cantores gospel houve correlação positiva forte entre o EASE- BR e o IFV nas subescalas de fadiga e limitação vocal (p<0,001), forte entre o EASE-BR e o IFV escore total (p<0,001), moderada entre o EASE-BR e o IFV na subescala de restrição vocal (p<0,001), moderada entre o IDV-10 e o IFV nas subescalas de fadiga e limitação vocal (p<0,001), moderada entre o IDV-10 e o IFV na subescala restrição vocal (p<0,001), fraca entre o IDV-10 e o IFV na subescala de desconforto físico associado a voz (p=0,012) e moderada entre o IDV-10 e o IFV escore total em cantores gospel (p<0,001).

Para o grupo CQ houve correlação positiva forte entre o EASE-BR e o IFV nas subescalas fadiga e limitação vocal (p<0,001) e forte entre o EASE-BR e o IFV escore total (p=0,002). Já para os sem queixa houve correlação positiva forte entre o EASE-BRe o IFV nas subescalas fadiga e limitação vocal (p<0,001), forte entre o EASE-BR e o IFV escore total (p<0,001), moderada entre o EASE-BR e o IFV na subescala restrição vocal (p=0,017) e moderada entre o IDV-10 e o IFV na subescala restrição vocal (p=0,017). A Tabela 2 sumariza a correlação entre o protocolo IFV e o EASE-BR e IDV-10.

**Tabela 2:** Correlação das variáveis EASE-Br e IDV-10 com a variável IFV em cantores gospel, cantores gospel com queixas vocais e cantores gospel sem queixas vocais

		Total		SQ		CQ	
		EASE-Br	IDV-10	EASE-Br	IDV-10	EASE-Br	IDV-10
IFV Fadiga e limitação vocal	r	0,948	0,545	0,931	0,154	0,888	0,379
	p-valor	<0,001*	<0,001*	<0,001^*^	0,399	<0,001*	0,250
IFV Restrição vocal	r	0,576	0,645	0,420	0,418	0,589	0,238
	p-valor	<0,001*	<0,001*	0,017*	0,017*	0,057	0,482
IFV Desconforto físico associado à voz	r	0,168	0,379	0,068	0,306	-0,166	0,555
	p-valor	0,283	0,012*	0,713	0,088	0,626	0,077
IFV Recuperação com repouso vocal	r	-0,081	0,085	-0,100	0,141	0,083	0,473
	p-valor	0,605	0,586	0,585	0,442	0,808	0,142
IFV total	r	0,887	0,537	0,840	0,134	0,817	0,414
	p-valor	<0,001*	<0,001*	<0,001*	0,466	0,002*	0,206

Teste de Correlação de Spearman

* variáveis estatisticamente significantes

**Legenda:** r=coeficiente de correlação

Houve diferença para os escores do IDV-10 subescalas fadiga e limitação vocal (U=148,5; p=0,045), EASE-BR (U=138; p=0,024) e IFV escore total (U=121,5; p=0,008), sendo que indivíduos do gênero feminino apresentaram valores maiores que os do gênero masculino. Já para o IFV subescala recuperação com repouso vocal, indivíduos do gênero masculino apresentaram valores maiores que os do gênero feminino (U=143; p=0,029), conforme a Tabela 3.

**Tabela 3:** Análise inferencial de comparação da variável EASE-Br, IFV e IDV-10 em função da variável gênero em cantores gospel

Variável	Gênero	Média	DP	Mediana	U	p-valor
IDV-10	Masculino	3,55	3,84	2,50	202,000	0,477
	Feminino	4,57	4,69	3,00		
EASE-Br	Masculino	16,77	9,31	17,00	138,000	0,024*
	Feminino	23,71	9,92	21,00		
IFV						
Fadiga e limitação vocal	Masculino	22,82	10,94	23,00	148,500	0,045^*^
	Feminino	30,48	12,25	31,00		
IFV						
Restrição vocal	Masculino	2,09	1,77	2,00	183,000	0,234
	Feminino	2,90	2,28	2,00		
IFV						
Desconforto físico associado à voz	Masculino	1,27	1,83	0,00	200,500	0,420
	Feminino	2,33	3,02	0,00		
IFV						
Recuperação com repouso vocal	Masculino	9,59	3,14	10,00	143,000	0,029*
	Feminino	7,05	4,12	8,00		
IFV						
Total	Masculino	28,59	12,38	26,50	121,500	0,008*
	Feminino	40,67	15,48	39,00		

Teste de Mann-Whitney

* variáveis estatisticamente significantes

Legenda: DP=desvio padrão; U= valor de U de Mann-Whitney

Nos indivíduos do grupo SQ, houve diferença para os escores do EASE-BR (U=73; p=0,044) e do IFV escore total (U=71; p=0,036), sendo que indivíduos do gênero feminino apresentaram valores maiores que indivíduos do gênero masculino. Já para o IFV subescala recuperação com repouso vocal, indivíduos do gênero masculino, apresentaram valores maiores que os do gênero feminino (U=75,5; p=0,049), conforme mostra a Tabela 4. Não foi encontrada diferença entre os escores dos protocolos e o gênero dos indivíduos do CQ.

**Tabela 4:** Análise inferencial de comparação da variável EASE-Br, IFV e IDV-10 em função da variável gênero em cantores gospel sem queixas vocais

Variável	Gênero	Média	DP	Mediana	U	p-valor
IDV-10	Masculino	2,06	2,18	1,50	122,500	0,892
	Feminino	1,64	1,28	1,50		
EASE-Br	Masculino	14,67	8,08	12,50	73,000	0,044^*^
	Feminino	21,21	9,66	17,50		
IFV						
Fadiga e limitação vocal	Masculino	19,94	9,28	19,50	87,000	0,138
	Feminino	25,36	10,02	22,00		
IFV						
Restrição vocal	Masculino	1,83	1,82	1,00	117,500	0,738
	Feminino	1,71	1,27	2,00		
IFV						
Desconforto físico associado à voz	Masculino	1,06	1,83	0,00	112,000	0,549
	Feminino	1,86	2,71	0,00		
IFV						
Recuperação com repouso vocal	Masculino	9,56	3,42	10,00	75,500	0,049*
	Feminino	6,64	4,48	7,00		
IFV						
Total	Masculino	25,28	10,18	25,00	71,000	0,036*
	Feminino	34,29	13,84	34,50		

Teste de Mann-Whitney

* variáveis estatisticamente significantes

**Legenda:** DP=desvio padrão; U= valor de U de Mann-Whitney

## DISCUSSÃO

Cantores amadores gospel muitas vezes atuam com alta demanda e sem orientação e preparação vocais adequadas, podendo sofrer com queixas de fadiga e desvantagem vocal^(3,5,6)^. O objetivo deste estudo foi investigar o impacto imediato na voz de cantores gospel com e sem queixa vocal após uma apresentação individual de uma hora.

Os dados de faixa etária e tempo de atuação no canto indicam que essa população começa as atividades de canto precocemente e de forma intuitiva, essencialmente autodidata. Cantores evangélicos cantam há muitos anos na igreja, podendo iniciar as atividades vocais em corais infantis e sem aulas de canto formais^(19)^.

A maioria dos participantes relataram serem profissionais da voz, tais como professores, jornalistas, músicos, líderes religiosos, dentre outros, entretanto, não relataram realizar alguma atividade para manutenção da saúde vocal como técnicas de aquecimento ou desaquecimento em suas atividades. Além da alta demanda vocal de canto na igreja esses cantores, por serem amadores, também podem ter alta demanda vocal no trabalho e não terem uma rotina de cuidado vocal adequada ou fazer consultas otorrinolaringológicas com frequência^(2,3,20)^.

Além disso, o fato de nenhum dos participantes ter buscado atendimento na área da voz pode evidenciar que essa queixa esteja sendo negligenciada. Esse tipo de falta de valorização de sintomas de problemas de voz também é comum entre outras populações de profissionais da voz como professores e teleoperadores^(21-24)^.

Embora ambos os grupos apresentassem médias do IFV acima da nota de corte, o grupo de cantores com queixa apresentou maiores escores neste protocolo, além de maior desvantagem vocal e maior dificuldade de cantar facilmente (Tabela 1). Além disso, os protocolos EASE-BR e IDV-10 evidenciaram que a rouquidão foi um sintoma prevalente nesse grupo que continua cantando apesar das queixas vocais, o que pode piorar sua condição laríngea. É interessante comentar que um estudo que avaliou a desvantagem vocal de 206 cantores populares evidenciou que os que apresentavam queixas vocais tiveram autopercepção de pior qualidade de vida e de maior desvantagem vocal^(25)^.

Especialmente para os cantores sem queixa vocal, as dificuldades relacionadas à habilidade de cantar estavam relacionadas à restrição ocasionada pela fadiga vocal, como uma provável consequência da elevada demanda vocal do canto gospel (Tabela 2). Dado semelhante foi observado em um ensaio controlado realizado com 30 cantores de coral adultos, evidenciando que essa população, apesar de não apresentar queixas vocais, as queixas aumentam conforme aumenta a sensação de fadiga^(26)^. Nos sujeitos sem queixa vocal, apesar de não terem pontuado acima da nota de corte do IDV-10, as queixas evidenciadas neste protocolo pareciam estar relacionadas com a restrição ocasionada pelos sintomas de fadiga vocal.

Para todos os grupos ficou evidente que os sintomas de fadiga vocal afetam diretamente a habilidade de cantar e podem contribuir para uma maior percepção de desvantagem vocal. Sabe-se que a fadiga vocal pode dificultar o canto modificando a tessitura da voz bem como suas regiões de passagem^(12)^. Tais dados evidenciam que essa população pode se beneficiar de orientações vocais voltadas para técnicas de aquecimento e desaquecimento vocal, visto que essas práticas adequam a musculatura vocal para as demandas e promovem uma produção vocal equilibrada^(27)^, além disso o desaquecimento, em específico, diminui a sobrecarga vocal e auxilia na remoção do ácido lático, evitando a dor e fadiga muscular^(28)^.

Ao se realizar uma análise por gênero evidenciada nas Tabelas 3 e 4, pode-se verificar que mulheres apresentam maior variação na habilidade ao cantar assim como desvantagens, ambas relacionadas aos sintomas de fadiga vocal. Já homens apresentam mais queixas relacionadas à recuperação da fadiga vocal. Isso pode ser explicado pela maior desvantagem vocal de mulheres em relação aos homens devido a características anatômicas e fisiológicas específicas do sexo feminino como o formato das cartilagens cricóidea e tireóidea que fazem com que muitas laringes femininas apresentem uma fenda fisiológica^(29)^. Já a fadiga vocal pode influenciar negativamente na habilidade de cantar^(12,26)^.

Considera-se uma limitação deste estudo a aplicação dos protocolos apenas imediatamente após a apresentação, impossibilitando uma comparação com queixas e sintomas que pudessem estar presentes previamente à performance. Além disso, questões quanto à saúde geral e aulas de canto também não foram levantadas. Não foi realizado cálculo amostral, deste modo as inferências evidenciadas neste estudo correspondem a este grupo estudado. Outro fato que é importante salientar é que para se separar cantores com e sem queixa vocal, foi utilizado o protocolo IDV-10, entretanto o Índice de Desvantagem Vocal para o Canto Moderno (IDCM)^(30)^ poderia levantar queixas específicas dessa população e comparar com a desvantagem vocal de cantores de outros estudos; contudo, esse instrumento ainda não apresenta nota de corte calculada. No entanto, algumas das queixas específicas da voz cantada puderam ser evidenciadas pelo protocolo EASE-BR.

Os resultados deste estudo têm implicações importantes para a prática clínica da Fonoaudiologia com cantores gospel. Os achados indicam que esses cantores, mesmo sem queixas vocais, podem apresentar sinais de fadiga e desvantagem vocal após a performance. Isso ressalta a importância de oferecer orientações vocais adequadas e programas de prevenção e promoção da saúde vocal para essa população. O uso de protocolos de autoavaliação vocal, como o EASE-BR, IDV-10 e o IFV, pode ser útil para identificar sinais de fadiga e desvantagem vocal e direcionar intervenções fonoaudiológicas.

## CONCLUSÃO

Cantores com queixa vocal apresentaram maiores índices de fadiga vocal, desvantagem vocal e maior dificuldade de cantar imediatamente após uma apresentação de uma hora. Cantores sem queixa vocal podem ter a habilidade de cantar prejudicada pelos sintomas de fadiga vocal. Variações na habilidade ao cantar e desvantagens vocais de cantores amadores gospel podem ter relação direta com a percepção de fadiga vocal.

São necessários mais estudos que investiguem a demanda específica dessa população e que possam sugerir estratégias de preparação vocal para esses indivíduos. Além disso, esses cantores se beneficiariam de medidas de promoção e prevenção de saúde vocal, visto que são autodidatas e possuem alta demanda vocal nas igrejas.
